# Quantitative assessment of coronary plaque volume change related to triglyceride glucose index: The Progression of AtheRosclerotic PlAque DetermIned by Computed TomoGraphic Angiography IMaging (PARADIGM) registry

**DOI:** 10.1186/s12933-020-01081-w

**Published:** 2020-07-18

**Authors:** Ki-Bum Won, Byoung Kwon Lee, Hyung-Bok Park, Ran Heo, Sang-Eun Lee, Asim Rizvi, Fay Y. Lin, Amit Kumar, Martin Hadamitzky, Yong-Jin Kim, Ji Min Sung, Edoardo Conte, Daniele Andreini, Gianluca Pontone, Matthew J. Budoff, Ilan Gottlieb, Eun Ju Chun, Filippo Cademartiri, Erica Maffei, Hugo Marques, Pedro de Araújo Gonçalves, Jonathon A. Leipsic, Sanghoon Shin, Jung Hyun Choi, Renu Virmani, Habib Samady, Kavitha Chinnaiyan, Gilbert L. Raff, Peter H. Stone, Daniel S. Berman, Jagat Narula, Leslee J. Shaw, Jeroen J. Bax, James K. Min, Hyuk-Jae Chang

**Affiliations:** 1 Department of Cardiology, Ulsan University Hospital, University of Ulsan College of Medicine, Ulsan, South Korea; 2 Department of Cardiology, Severance Cardiovascular Hospital, Yonsei-Cedars-Sinai Integrative Cardiovascular Imaging Research Center, Yonsei University College of Medicine, Yonsei University Health System, 50-1 Yonsei-ro, Seodaemun-gu, Seoul, 03722 South Korea; 3 Department of Cardiology, Gangnam Severance Hospital, Yonsei University College of Medicine, Seoul, South Korea; 4 Yonsei-Cedars-Sinai Integrative Cardiovascular Imaging Research Center, Yonsei University College of Medicine, Yonsei University Health System, Seoul, South Korea; 5 Department of Cardiology, Catholic Kwandong University International St. Mary’s Hospital, Incheon, South Korea; 6 Department of Cardiology, Hanyang University Seoul Hospital, Hanyang University College of Medicine, Seoul, South Korea; 7 Department of Cardiology, Ewha Womans University Medical Center, Seoul, South Korea; 8 Department of Radiology, New York-Presbyterian Hospital and Weill Cornell Medical College, New York, NY USA; 9 Department of Radiology, Mayo Clinic, Rochester, MN USA; 10 Department of Radiology and Nuclear Medicine, German Heart Center Munich, Munich, Germany; 11 Department of Cardiology, Seoul National University Hospital, Seoul, South Korea; 12 Centro Cardiologico Monzino, IRCCS, Milan, Italy; 13 Department of Medicine, Los Angeles Biomedical Research Institute, Torrance, CA USA; 14 Department of Radiology, Casa de Saude São Jose, Rio de Janeiro, Brazil; 15 Department of Radiology, Seoul National University Bundang Hospital, Sungnam, South Korea; 16 Cardiovascular Imaging Center, SDN IRCCS, Naples, Italy; 17 Department of Radiology, Area Vasta 1/ASUR, Marche, Urbino, Italy; 18 UNICA, Unit of Cardiovascular Imaging, Hospital da Luz, Lisbon, Portugal; 19 Department of Medicine and Radiology, University of British Columbia, Vancouver, BC Canada; 20 Department of Cardiology, Busan University Hospital, Busan, South Korea; 21 Department of Pathology, CVPath Institute, Gaithersburg, MD USA; 22 Department of Cardiology, Emory University School of Medicine, Atlanta, GA USA; 23 Department of Cardiology, William Beaumont Hospital, Royal Oak, MI USA; 24 Department of Cardiovascular Medicine, Brigham and Women’s Hospital, Harvard Medical School, Boston, MA USA; 25 Department of Imaging and Medicine, Cedars Sinai Medical Center, Los Angeles, CA USA; 26 Icahn School of Medicine at Mount Sinai, Mount Sinai Heart, New York, USA; 27 Zena and Michael A. Wiener Cardiovascular Institute, and Marie-Josee and Henry R. Kravis Center for Cardiovascular Health, New York, NY USA; 28 Department of Cardiology, Leiden University Medical Center, Leiden, The Netherlands

**Keywords:** Triglyceride glucose index, Coronary artery disease, Atherosclerosis, Coronary computed tomography angiography

## Abstract

**Background:**

The association between triglyceride glucose (TyG) index and coronary atherosclerotic change remains unclear. We aimed to evaluate the association between TyG index and coronary plaque progression (PP) using serial coronary computed tomography angiography (CCTA).

**Methods:**

A total of 1143 subjects (aged 60.7 ± 9.3 years, 54.6% male) who underwent serial CCTA with available data on TyG index and diabetic status were analyzed from The Progression of AtheRosclerotic PlAque DetermIned by Computed TomoGraphic Angiography IMaging (PARADIGM) registry. PP was defined as plaque volume (PV) (mm^3^) at follow-up minus PV at index > 0. Annual change of PV (mm^3^/year) was defined as PV change divided by inter-scan period. Rapid PP was defined as the progression of percent atheroma volume (PV divided by vessel volume multiplied by 100) ≥ 1.0%/year.

**Results:**

The median inter-scan period was 3.2 (range 2.6–4.4) years. All participants were stratified into three groups based on TyG index tertiles. The overall incidence of PP was 77.3%. Baseline total PV (group I [lowest]: 30.8 (0.0–117.7), group II: 47.2 (6.2–160.4), and group III [highest]: 57.5 (8.4–154.3); P < 0.001) and the annual change of total PV (group I: 5.7 (0.0–20.2), group II: 7.6 (0.5–23.5), and group III: 9.4 (1.4–27.7); P = 0.010) were different among all groups. The risk of PP (odds ratio [OR] 1.648; 95% confidence interval [CI] 1.167–2.327; P = 0.005) and rapid PP (OR 1.777; 95% CI 1.288–2.451; P < 0.001) was increased in group III compared to that in group I. TyG index had a positive and significant association with an increased risk of PP and rapid PP after adjusting for confounding factors.

**Conclusion:**

TyG index is an independent predictive marker for the progression of coronary atherosclerosis.

*Clinical registration* ClinicalTrials.gov NCT02803411

## Background

Coronary artery disease (CAD) is a leading cause of morbidity and mortality worldwide [1]. It is important to understand the coronary atherosclerotic progression for the prevention of adverse cardiovascular (CV) events. Numerous previous studies have suggested the significant role of insulin resistance (IR) in the development of CAD [2–4]. Recently, the triglyceride glucose (TyG) index has been suggested to be a reliable surrogate marker of IR [5–7]. Several cross-sectional studies have reported that TyG index is associated with CAD, especially with coronary artery calcification (CAC) [8, 9]. However, longitudinal data on the association between TyG index and coronary plaque progression (PP) is scarce. Coronary computed tomography angiography (CCTA) is a well-established non-invasive imaging tool with high diagnostic performance for coronary atherosclerosis and predictive value for adverse CV events [10–13]. Therefore, we aimed to examine the association between baseline TyG index and coronary PP using serial CCTA.

## Methods

### Study design and populations

The Progression of AtheRosclerotic PlAque DetermIned by Computed TomoGraphic Angiography IMaging (PARADIGM) is a prospective, international, and multicenter observational registry designed to evaluate associations between clinical variables and coronary atherosclerotic changes using serial CCTA [14]. Between 2003 and 2015, 2252 consecutive subjects underwent serial CCTA at 13 centers in 7 countries. Among these subjects, 1143 subjects with available information on TyG index and diabetic status were included in the present study. The characteristics of coronary plaques in all participants were categorized based on the TyG index tertile. TyG index was calculated as ln [fasting triglycerides (mg/dL) × fasting glucose (mg/dL)/2]. Diabetes was defined as treatment with oral hypoglycemic agent or insulin or fasting blood glucose (FBG) ≥ 126 mg/dL. The institutional review boards approved this study at each site.

### Acquisition and interpretation of CCTA

**Fig. 1:**
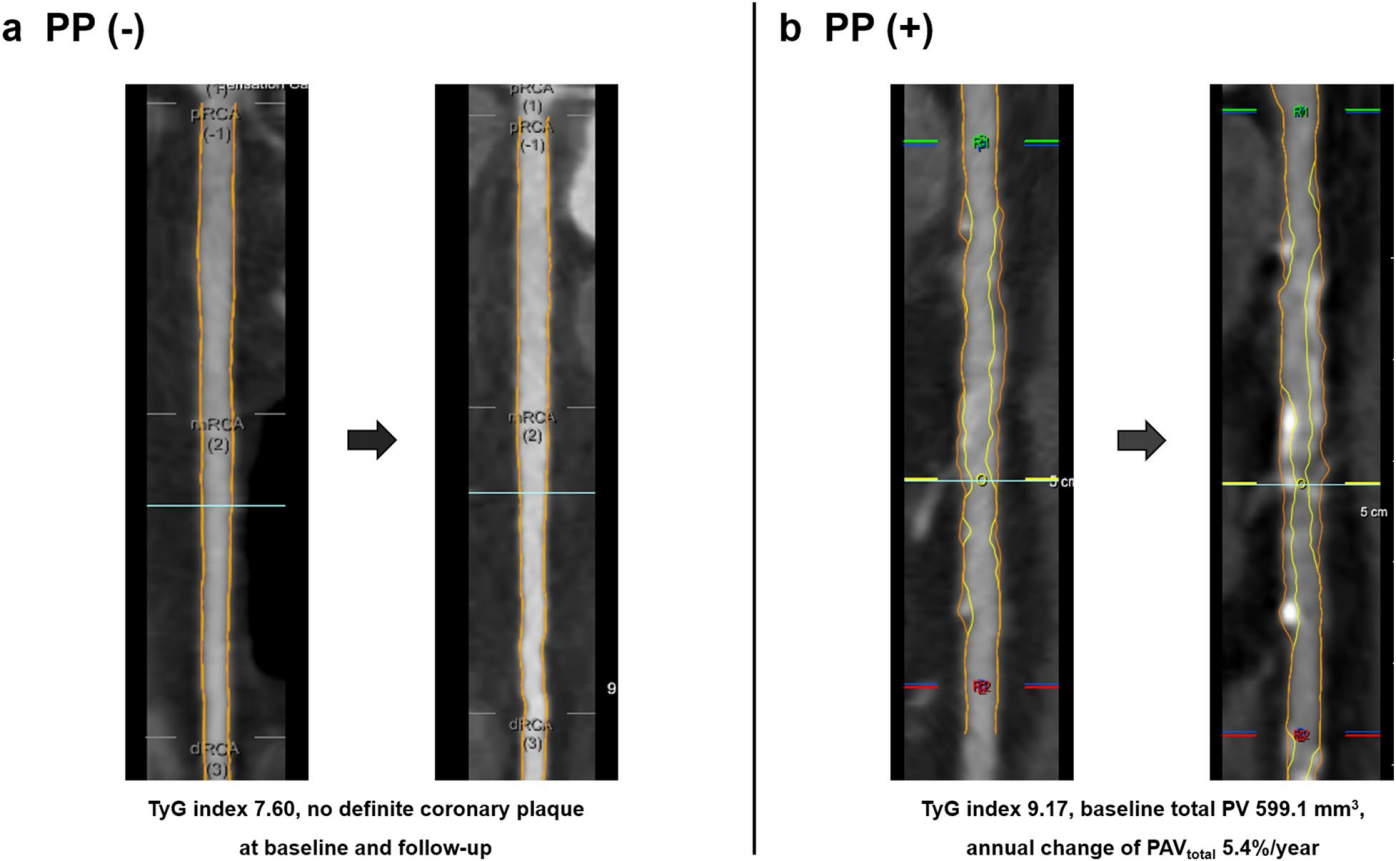
Representative CCTA images. *CCTA* coronary computed tomography angiography, *TyG* triglyceride glucose

All data acquisition and post-processing of CCTA were in accordance with the Society of Cardiovascular Computed Tomography guidelines [15, 16]. CCTA was performed with a ≥ 64-detector row scanner at all centers. All datasets from each center were transferred to an offline workstation for analysis with a semi-automated plaque analysis software (QAngioCT Research Edition v2.1.9.1; Medis Medical Imaging Systems, Leiden, the Netherlands) using manual correction. Segments with diameter ≥ 2 mm were evaluated using a modified 17-segment American Heart Association model [16]. Regardless of the presence of atherosclerotic plaques, plaque volume (PV) (mm^3^) of every coronary segment was obtained and summated to generate total PV per patient. Coronary plaques were further classified by composition according to the pre-defined intensity cut-offs in Hounsfield units (HU) for calcified plaques (≥ 351 HU), fibrous plaques (131–350 HU), fibro-fatty plaques (31–130 HU), and necrotic cores (-30 to 30 HU) [17, 18]. For comparing longitudinal CCTA images, all baseline and follow-up coronary segments were registered together with fiduciary landmarks, including the distance from the ostia or branch vessel take-offs. PV change was defined as plaque volume at follow-up CCTA minus plaque volume at baseline CCTA. Annual change of PV (mm^3^/year) was defined as total PV change divided by inter-scan period. Moreover, normalized total atheroma volume (TAV_norm_) (mm^3^) was defined as total PV divided by vessel length, multiplied by the mean participants’ vessel length. Annual change of TAV_norm_ (mm^3^/year) was defined as TAV_norm_ divided by the inter-scan period. While total percent atheroma volume (PAV_total_) (%) was defined as PV divided by vessel volume, multiplied by 100, annual change of PAV_total_ (%/year) was defined as total PAV divided by inter-scan period, and plaque progression (PP) was defined as the difference in plaque volume between follow-up and baseline CCTA > 0. Further, rapid PP (%/year) was defined as an annual progression of PAV ≥ 1.0% [19, 20]. Representative CCTA images are presented in Fig. 1.

### Statistical analysis

Continuous variables are expressed as mean ± SD or medians and interquartile range, while categorical variables are presented as absolute values and proportions. Continuous variables were compared using an independent *t* test or the Mann–Whitney U-test, as appropriate and categorical variables were compared using the χ^2^-test or Fisher’s exact test, as appropriate. Coronary characteristics across TyG index tertiles were compared using one-way analysis of variance or the Kruskal–Wallis test for continuous variables, as appropriate. Univariate logistic regression analysis was performed to evaluate the association between clinical variables and coronary PP. Further, multivariate logistic regression analyses were performed to identify the independent impact of TyG index on coronary PP. Variables with P < 0.05 in the univariate logistic regression analysis were considered confounding variables and entered into the multivariate logistic regression models, except the individual component of TyG index. All statistical analyses were performed using the Statistical Package for the Social Sciences version 19 (SPSS, Chicago, Illinois). A P value < 0.05 was considered statistically significant for all analyses.

## Results

### Baseline characteristics

The mean age of the 1143 participants (624 male, 54.6%) was 60.7 ± 9.3 years. Median inter-scan period was 3.2 (range, 2.6–4.4) years. Coronary PP was observed in 883 (77.3%) participants during follow-up. The clinical characteristics of participants according to PP are presented in Table 1. Age, systolic blood pressure (BP), body mass index (BMI), serum triglyceride and FBG levels, prevalence of male sex, hypertension, diabetes, hyperlipidemia, and the use of aspirin, angiotensin-converting enzyme inhibitor (ACEI)/angiotensin receptor blocker (ARB), and statin were significantly higher in subjects with PP than in those without it. Subjects with PP had significantly lower levels of high-density lipoprotein cholesterol (HDL-C) than those without PP. TyG index values were higher in subjects with PP than in those without it (8.85 ± 0.60 vs. 8.69 ± 0.55; P < 0.001).

**Table 1:** Baseline characteristics

	Total (n = 1143)	PP (−) (n = 260)	PP (+) (n = 883)	P
Age, years	60.7 ± 9.3	58.7 ± 9.6	61.3 ± 9.1	< 0.001
Male, n (%)	624 (54.6)	122 (46.9)	502 (56.9)	0.005
Systolic BP, mmHg	126.1 ± 16.6	123.7 ± 16.3	126.8 ± 16.6	0.011
Diastolic BP, mmHg	77.0 ± 10.6	75.9 ± 11.1	77.3 ± 10.5	0.079
BMI, kg/m^2^	24.8 ± 3.0	24.3 ± 3.0	25.0 ± 3.0	0.002
BMI ≥ 25.0 kg/m^2^, n (%)	493 (43.9)	97 (37.5)	396 (45.8)	0.017
Hypertension, n (%)	674 (59.0)	123 (47.3)	551 (62.5)	< 0.001
Diabetes, n (%)	319 (27.9)	48 (18.5)	271 (30.7)	< 0.001
Hyperlipidemia, n (%)	381 (33.3)	69 (26.5)	312 (35.4)	0.008
Current smoking, n (%)	213 (18.6)	42 (16.2)	171 (19.4)	0.239
Medications, n (%)				
Aspirin	555 (48.6)	107 (41.2)	448 (50.8)	0.006
Beta blocker	353 (31.0)	81 (31.2)	272 (30.9)	0.940
ACEI/ARB	389 (34.2)	67 (25.9)	332 (36.6)	0.001
Statin	520 (45.5)	89 (34.2)	431 (49.5)	< 0.001
Insulin therapy	33 (2.9)	5 (2.0)	28 (3.2)	0.294
Total cholesterol, mg/dL	182.6 ± 38.4	184.4 ± 39.6	182.0 ± 38.1	0.393
Triglyceride, mg/dL	143.3 ± 83.1	132.8 ± 73.9	146.4 ± 85.4	0.020
HDL-C, mg/dL	48.7 ± 12.3	50.3 ± 12.8	48.2 ± 12.1	0.016
LDL-C, mg/dL	111.5 ± 34.1	113.5 ± 34.2	110.9 ± 34.0	0.289
Creatinine, mg/dL	1.01 ± 0.67	0.96 ± 0.47	1.01 ± 0.72	0.233
FBG, mg/dL	110.7 ± 35.0	104.2 ± 28.6	112.7 ± 36.5	< 0.001
HbA1c, %	6.46 ± 1.27	6.26 ± 1.19	6.51 ± 1.28	0.067
TyG index	8.81 ± 0.59	8.69 ± 0.55	8.85 ± 0.60	< 0.001

Values are presented as mean ± standard deviation or number (%)

*ACEI* angiotensin-converting enzyme inhibitor, *ARB* angiotensin receptor blocker, *BMI* body mass index, *BP* blood pressure, *FBG* fasting blood glucose, *HDL-C* high-density lipoprotein cholesterol, *LDL-C* low-density lipoprotein cholesterol, *TyG* triglyceride glucose

### Comparison of baseline PV and annual change of PV according to TyG index tertile

Baseline total PV (mm^3^) was as follows: group I [lowest]: 30.8 (0.0–117.7), group II: 47.2 (6.2–160.4), and group III [highest]: 57.5 (8.4–154.3), P < 0.001. Baseline TAV_norm_ values were as follows: group I, 33.0 (0.0–122.3); group II, 54.1 (7.4–192.3); and group III, 61.2 (9.5–165.6); P = 0.001. PAV_total_ was as follows: group I, 1.6 (0.0–6.1); group II: 2.8 (0.4–8.8); and group III: 3.0 (0.5–8.1); P = 0.001. There were significant differences among the TyG index tertile groups at baseline. Regarding coronary plaque subtypes, there was a significant difference in the fibrous, fibrous-fatty, necrotic-core, and dense calcium PVs among all groups at baseline. During follow-up, the annual change of the total PV was as follows: group I, 5.7 (0.0–20.2); group II, 7.6 (0.5–23.5); and group III, 9.4 (1.4–27.7); P = 0.0101; 2) and of TAV_norm_ was as follows: group I, 6.2 (0.0–19.9); group II, 7.8 (0.5–25.4); and group III, 9.3 (1.7–31.2); P = 0.005. PAV_total_ [group I: 0.3 (0.0–0.9), group II: 0.4 (0.0–1.3), and group III: 0.5 (0.1–1.4); P = 0.006] was different among all the groups. There was a significant difference in the annual change of fibrous and dense calcium PVs (Table 2).

**Table 2:** Baseline and changes in the coronary plaque characteristics

	Total (n = 1143)	TyG index tertiles			P
		I (lowest) 7.20–8.53 (n = 382)	II 8.54–9.02 (n = 388)	III (highest) 9.03–10.84 (n = 373)	
At baseline					
Plaque volume (mm^3^)					
Total	44.1 (3.5–139.6)	30.8 (0.0–117.7)	47.2 (6.2 −160.4)	57.5 (8.4–154.3)	< 0.001
Fibrous	20.3 (0.8–59.1)	13.7 (0.0–49.7)	22.5 (2.9–67.3)	24.3 (3.8–64.4)	0.001
Fibrous-fatty	3.5 (0.0–23.4)	1.4 (0.0–14.8)	3.6 (0.0–25.3)	6.1 (0.0–31.8)	< 0.001
Necrotic-core	0.0 (0.0–1.5)	0.0 (0.0–0.7)	0.0 (0.0–1.6)	0.1 (0.0–2.4)	< 0.001
Dense calcium	6.4 (0.0–38.1)	2.8 (0.0–33.5)	8.0 (0.0–41.2)	8.3 (0.0–36.5)	0.027
TAV_norm_ (mm^3^)	47.6 (2.6–151.5)	33.0 (0.0–122.3)	54.1 (7.4–192.3)	61.2 (9.5–165.6)	0.001
PAV_total_ (%)	2.5 (0.1–7.7)	1.6 (0.0–6.1)	2.8 (0.4–8.8)	3.0 (0.5–8.1)	0.001
Annual change					
Plaque volume (mm^3^/year)					
Total	7.6 (0.5–22.2)	5.7 (0.0–20.2)	7.6 (0.5–23.5)	9.4 (1.4–27.7)	0.010
Fibrous	1.9 (0.0–8.6)	1.1 (0.0–6.9)	1.9 (0.0–8.7)	2.8 (0.0–9.8)	0.022
Fibrous-fatty	0.0 (-0.8–1.4)	0.0 (-0.5–0.9)	0.0 (-0.9–1.3)	0.0 (-1.0–2.3)	0.341
Necrotic-core	0.0 (0.0–0.1)	0.0 (0.0–0.0)	0.0 (0.0–0.1)	0.0 (-0.1–0.1)	0.659
Dense calcium	3.4 (0.1–11.7)	2.1 (0.0–8.7)	3.7 (0.3–12.1)	3.9 (0.4–13.3)	0.016
TAV_norm_ (mm^3^/year)	7.7 (0.4–24.4)	6.2 (0.0–19.9)	7.8 (0.5–25.4)	9.3 (1.7–31.2)	0.005
PAV_total_ (%/year)	0.4 (0.0–1.2)	0.3 (0.0–0.9)	0.4 (0.0–1.3)	0.5 (0.1–1.4)	0.006

Values are presented as median (interquartile range)

*PAV*_*total*_ total percent atheroma volume, *TAV*_*norm*_ normalized total atheroma volume, *TyG* triglyceride glucose

### Association of clinical variables with coronary atherosclerotic change

Age (odds ratio [OR] 1.031; 95% confidence interval [CI] 1.016–1.047; P < 0.001), male sex (OR 1.490; 95% CI 1.129–1.967; P = 0.005), systolic BP (OR 1.012; 95% CI 1.003–1.022; P = 0.012), BMI (OR 1.077; 95% CI 1.026–1.130; P = 0.003), and HDL-C (OR 0.987; 95% CI 0.976–0.998; P = 0.017) were associated with coronary PP. Among the TyG tertile groups, PP risk was increased in group III compared with that in group I (OR 1.648; 95% CI 1.167–2.327; P = 0.005) (Table 3).

**Table 3:** Univariate logistic regression analysis for the association of clinical variables with the risk of coronary PP

Variables	OR (95% CI)	P
Age, per 1 year	1.031 (1.016–1.047)	< 0.001
Male	1.490 (1.129–1.967)	0.005
Systolic BP, per 1 mmHg	1.012 (1.003–1.022)	0.012
Diastolic BP, per 1 mmHg	1.013 (0.999–1.027)	0.079
BMI, per 1 kg/m^2^	1.077 (1.026–1.130)	0.003
Total cholesterol, per 1 mg/dL	0.998 (0.995–1.002)	0.393
Triglyceride, per 1 mg/dL	1.002 (1.000–1.004)	0.021
HDL-C, per 1 mg/dL	0.987 (0.976–0.998)	0.017
LDL-C, per 1 mg/dL	0.998 (0.994–1.002)	0.288
FBG, per 1 mg/dL	1.009 (1.004–1.014)	< 0.001
HbA1c, per 1%	1.198 (0.986–1.456)	0.069
TyG index tertiles		
I (lowest)	1	–
II	1.294 (0.932–1.797)	0.123
III (highest)	1.648 (1.167–2.327)	0.005

*BMI* body mass index, *BP* blood pressure, *CI* confidence interval, *FBG* fasting blood glucose, *HbA1c* hemoglobin A1c; HDL-C, high-density lipoprotein cholesterol; LDL-C, low-density lipoprotein cholesterol; OR, odds ratio; TyG, triglyceride glucose

### Subgroup analysis for the relationship of TyG index with coronary PP

**Fig. 2:**
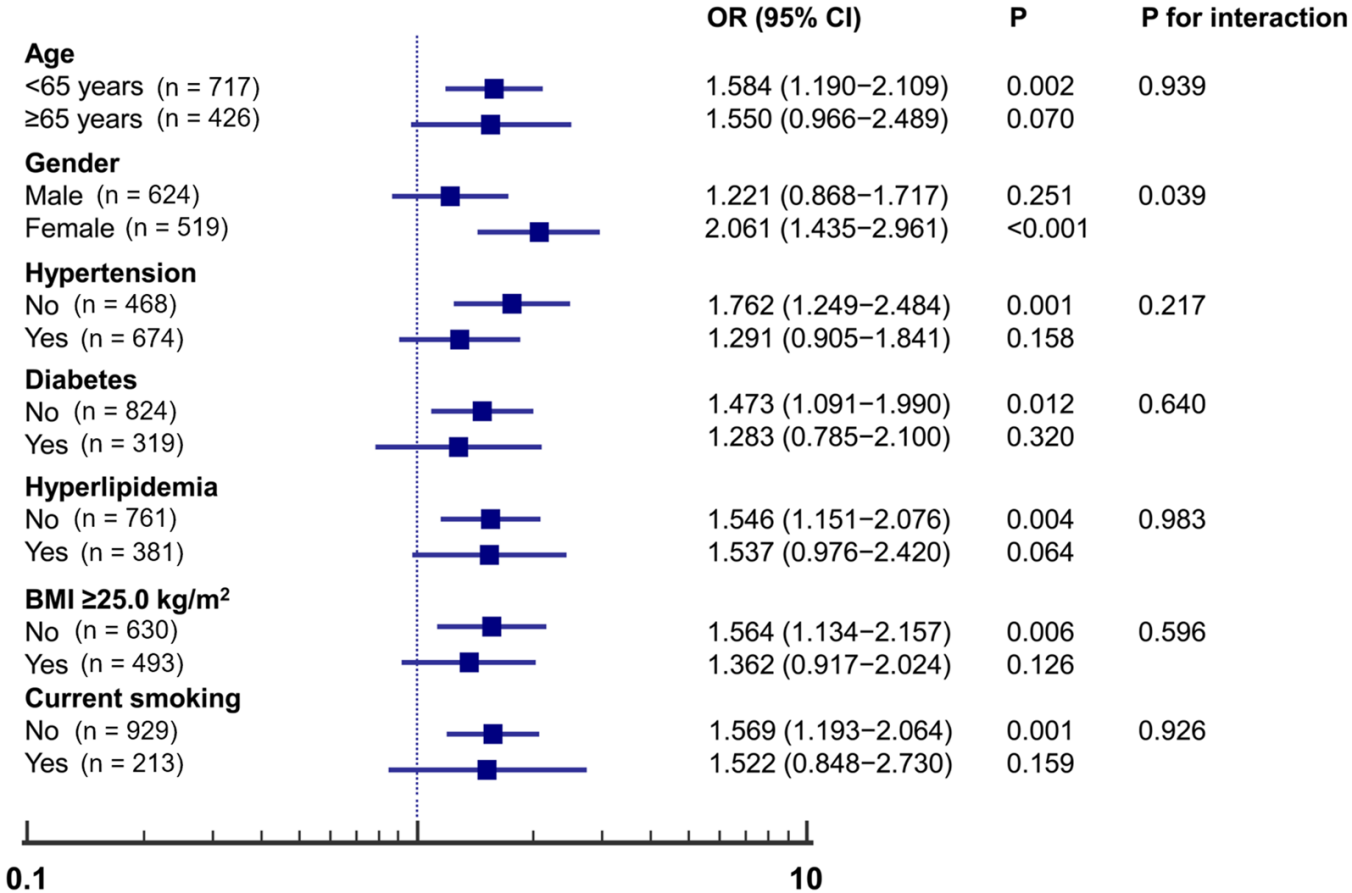
Subgroup analysis for the impact of TyG index on coronary PP. *TyG* triglyceride glucose, *PP* plaque progression

Figure 2 shows the subgroup analysis of the estimated OR of TyG index for coronary PP. The TyG index was significantly associated with an increased risk of PP in subgroups of aged < 65 years (OR 1.584; 95% CI 1.190–2.109; P = 0.002), females (OR 2.061; 95% CI 1.435–2.961; P < 0.001), as well as those without hypertension (OR 1.762; 95% CI 1.249–2.484; P = 0.001), and diabetes (OR 1.473; 95% CI 1.091–1.990; P = 0.012). The same association was observed with hyperlipidemia (OR 1.546; 95% CI 1.151–2.076; P = 0.004), BMI ≥ 25.0 kg/m^2^ (OR 1.564; 95% CI 1.134–2.157; P = 0.006), and current smoking status (OR 1.569; 95% CI 1.193–2.064; P = 0.001).

### TyG index on the risk of coronary PP

The results of multiple logistic regression models for the association between TyG index and PP risk are presented in Table 4. Increased TyG index values were significantly related to an increased risk of PP after adjusting for other confounding variables. After adjusting for traditional CV risk factors, TyG index was associated with coronary PP (OR 1.308; 95% CI 1.004–1.703; P = 0.046) (Additional file 1: Table S1). TyG index was particularly associated with the calcified PP among coronary plaque sub-types (Additional file 2: Table S2). Regarding rapid coronary PP, multivariate logistic regression analysis showed that the risk of rapid PP was increased in group III (OR 1.557; 95% CI 1.109–2.185; P = 0.011) compared with group I (Table 5).

**Table 4:** Multiple logistic models for the impact of TyG index on coronary PP

Variables	OR (95% CI)	P	RR (95% CI)	P
TyG index, per 1-unit increase				
Model 1	1.575 (1.232–2.015)	< 0.001	1.103 (1.049–1.160)	< 0.001
Model 2	1.598 (1.250–2.042)	< 0.001	1.111 (1.056–1.169)	< 0.001
Model 3	1.409 (1.062–1.869)	0.017	1.083 (1.021–1.150)	0.008

*BMI* body mass index, *BP* blood pressure, *CI* confidence interval, *HDL-C* high-density lipoprotein cholesterol, *OR* odds ratio, *PP* plaque progression, *RR* relative risk, *TyG* triglyceride glucose

Model 1: Unadjusted

Model 2: Adjusted for age and sex

Model 3: Adjusted for age, sex, systolic BP, BMI, and HDL-C

**Table 5:** Association of TyG index and traditional risk factors with rapid PP

Variables	Univariate		Multivariate	
	OR (95% CI)	P	OR (95% CI)	P
Age ≥ 65 years	1.836 (1.412–2.387)	< 0.001	1.717 (1.309–2.253)	< 0.001
Male	1.248 (0.962–1.620)	0.095		
Hypertension	1.561 (1.193–2.044)	0.001	1.292 (0.976–1.710)	0.074
Diabetes	1.845 (1.399–2.432)	< 0.001	1.509 (1.125–2.023)	0.006
Hyperlipidemia	1.468 (1.124–1.919)	0.005	1.340 (1.019–1.763)	0.036
BMI ≥ 25.0 kg/m^2^	1.120 (0.863–1.454)	0.393		
TyG index tertiles				
I (lowest)	1	–	1	–
II	1.362 (0.983–1.887)	0.063	1.242 (0.890–1.734)	0.202
III (highest)	1.777 (1.288–2.451)	< 0.001	1.557 (1.109–2.185)	0.011

BMI, body mass index; CI, confidence interval; CV, cardiovascular; OR, odds ratio; PP, plaque progression; TyG, triglyceride glucose

## Discussion

### Main findings

To the best our knowledge, this is first study to evaluate the longitudinal quantitative changes of coronary plaques and their subtypes related to TyG index using serial CCTA. This study identified a significant association between TyG index and coronary atherosclerosis progression. Previous cross-sectional studies have reported a significant relationship between TyG index and CAC prevalence [8, 9]. A recent longitudinal study revealed that elevated TyG index is independently associated with CAC progression [21]. However, this study had a retrospective design and included only a Korean population, which were limitations. Additionally, considering that non-calcified plaques might be related to an increased risk of acute coronary syndrome events [22], it might be important to compare longitudinal changes of non-calcified plaques according to TyG index values. In the present PARADIGM study, which had a prospective, international, and observational design, we identified that the baseline total PV and all subtypes as well as annualized change in total, fibrous, and dense-calcium PV increased with increasing TyG index values. In addition, the TyG index had a positive association with the annual change of total PV, TAV_norm_, and PAV_total_ (Additional file 3: Table S3). Even after adjusting for confounding factors, TyG index was related to the increased risk of PP as well as rapid PP. Regarding coronary plaque sub-types, TyG index was found to be associated with calcified PP after adjusting for traditional CV risk factors in a previous cross-sectional cohort study [9].

### Recent investigations on the longitudinal assessment of coronary atherosclerosis

To understand that the coronary atherosclerotic change is an important issue in clinical practice, it is well-known that diabetes has close association with the prevalence and severity of CCTA verified CAD progression [23]. Even asymptomatic diabetic patients experience plaque progression as well as evolution to overt or silent CAD, and an increase in the PV was reported to be associated with subsequent CV events [24]. In addition, the increased duration of diabetes combined with higher HbA1c levels deleteriously influences culprit-plaque characteristics among diabetic patients who suffered acute myocardial infarction [25]. A rapid plaque progression was specially observed in male patients and in patients with typical angina [26]. While helical flow in coronary arteries has a protective role against atherosclerotic wall thickness growth [27], an intrinsic calcification angle, defined as the angle externally projected by a vascular calcification, is a novel feature of coronary plaque vulnerability and its impact on fibrous cap stress is potentiated in more superficial calcifications, adding to the destabilizing role of smaller calcifications [28].

### Focused issue for the significance of TyG index

It is well-established that IR is a main mechanism in the development of type 2 diabetes. A previous PARADIGM study identified that individuals with established diabetes experienced greater PP, particularly, significantly greater progression of adverse plaque formation than those without diabetes [29]. In addition, unlike diabetes, pre-diabetic condition was not independently associated with coronary PP in the sub-study of same registry [30]; however, although pre-diabetes was defined according to the criteria used in previous studies, glycemic status was assessed based on only the levels of FBG and HbA1c without considering IR status among non-diabetic participants. According to the results of a recent large cross-sectional cohort study [31], TyG index had an independent and positive association with the risk of CAD and obstructive CAD in non-diabetic individuals; however, glycemic control status reflected in HbA1c rather than IR parameters was significantly related to the risk of both CAD and obstructive CAD in individuals with established diabetes. These results might support the hypothesis for the different pathogenesis of CAD according to diabetic status. In clinical practice, atherosclerosis-related adverse events commonly occurred even in people with low CV risk burden [32–34]. Thus, early detection of the presence and progression of subclinical atherosclerosis in this population is important. Recent studies have focused on defining useful predictors for subclinical atherosclerosis in individuals with low CV risk [35, 36]. Interestingly, although the statistical significance could be influenced by the sample size of the individual subgroup, this study showed that TyG index had a significant predictive value for PP in individuals without the traditionally known CV risk factors, especially in female subgroup. This result suggests that TyG index is a potential surrogate marker for the early detection of subclinical atherosclerosis in the absence of CV risk factors as reported in a recent cross-sectional cohort study [37]. Considering the pivotal role of IR in atherosclerosis progression by promoting apoptosis of macrophages, endothelial cells, and vascular smooth muscle cells [38–40], further prospective studies with larger sample sizes will be necessary to address the predictive value of TyG index for subclinical atherosclerosis in individuals with low CV risk burden.

### Limitations

There are some limitations in the present study. First, we only evaluated the association between baseline TyG index and coronary atherosclerotic change; longitudinal consecutive changes of TyG index during follow-up could not be confirmed. Second, the effects of anti-hypertensive and anti-diabetic medications were not controlled for because of the observational nature of the study design. Third, the homeostatic model assessment of insulin resistance was not analyzed and compared with TyG index because insulin levels were not measured in the PARADIGM registry. Fourth, we could not confirm the TyG index of the small coronary arteries in the present study. Fifth, a selection bias might be present because of the retrospective inclusion of participants. In addition, the results of CCTA at baseline could affect the performance of follow-up CCTA. Finally, despite our application of strict and standardized criteria for assessing CCTA, atherosclerotic findings can be affected by HU density. Despite these limitations, this study used serial CCTA to estimate coronary PVC and PP according to TyG index values in a large multicultural cohort subjects.

## Conclusion

The present study demonstrates the independent association between TyG index values and coronary PP based on serial quantitative assessment by CCTA during a relatively short-term period. Further large prospective and randomized studies with longer follow-up durations are necessary to confirm the results of the present study.

## Supplementary information

**Additional file 1: Table S1.** Clinical variables and annualized total PVC.

**Additional file 2: Table S2.** Multivariate logistic regression analysis for the association of clinical variables with coronary plaque progression.

**Additional file 3: Table S3.** Association of clinical variables with the annual change of total PV, TAV_norm_, and PAV_total_.

## Data Availability

The datasets used and analyzed during the current study are available from the corresponding author on reasonable request.

## Abbreviations

ACEI: Angiotensin-converting enzyme inhibitor
ARB: Angiotensin receptor blocker
BMI: Body mass index
BP: Blood pressure
CV: Cardiovascular
CAC: Coronary artery calcification
CAD: Coronary artery disease
CCTA: Coronary computed tomography angiography
CI: Confidence interval
FBG: Fasting blood glucose
HbA1c: Hemoglobin A1c
HDL-C: High-density lipoprotein cholesterol
IR: Insulin resistance
LCL-C: Low-density lipoprotein cholesterol
OR: Odds ratio
PVC: Plaque volume change
RR: Relative risk
PARADIGM: Progression of AtheRosclerotic PlAque DetermIned by Computed TomoGraphic Angiography IMaging
PAV_total_: Total percent atheroma volume
PP: Plaque progression
TAV_norm_: Normalized total atheroma volume
TyG: Triglyceride glucose
