# Supplementary material for: Quantitative assessment of coronary plaque volume change related to triglyceride glucose index: The Progression of AtheRosclerotic PlAque DetermIned by Computed TomoGraphic Angiography IMaging (PARADIGM) registry
Source: Cardiovasc Diabetol. 2020 Jul 18;19:113. doi: 10.1186/s12933-020-01081-w (PMC7368987; doi:10.1186/s12933-020-01081-w)
Supplement: Supplementary file 1 — Additional file 1: Table S1. Clinical variables and annualized total PVC. [file 12933_2020_1081_MOESM1_ESM.docx]

**Additional Table S1.** Association of clinical variables with coronary plaque progression

|  | Univariate | | Multivariate | |
| --- | --- | --- | --- | --- |
| Variables | OR (95% CI) | p | OR (95% CI) | p |
| Age, per 1 year | 1.031 (1.016–1.047) | <0.001 | 1.029 (1.012–1.046) | 0.001 |
| Male | 1.501 (1.138–1.979) | 0.004 | 1.630 (1.221–2.176) | 0.001 |
| Hypertension | 1.854 (1.403–2.451) | <0.001 | 1.441 (1.067–1.945) | 0.017 |
| Diabetes | 1.956 (1.386–2.760) | <0.001 | 1.481 (1.025–2.139) | 0.037 |
| Hyperlipidemia | 1.515 (1.114–2.062) | 0.008 | 1.367 (0.993–1.884) | 0.056 |
| BMI ≥25.0 kg/m^2^ | 1.413 (1.063–1.879) | 0.017 | 1.289 (0.954–1.741) | 0.098 |
| TyG index, per 1-increase | 1.575 (1.232–2.015) | <0.001 | 1.317 (1.013–1.713) | <0.040 |

CI, confidence interval; CV, cardiovascular; OR, odds ratio; TyG, triglyceride glucose
