# Supplementary material for: Quantitative assessment of coronary plaque volume change related to triglyceride glucose index: The Progression of AtheRosclerotic PlAque DetermIned by Computed TomoGraphic Angiography IMaging (PARADIGM) registry
Source: Cardiovasc Diabetol. 2020 Jul 18;19:113. doi: 10.1186/s12933-020-01081-w (PMC7368987; doi:10.1186/s12933-020-01081-w)
Supplement: Supplementary file 2 — Additional file 2: Table S2. Multivariate logistic regression analysis for the association of clinical variables with coronary plaque progression. [file 12933_2020_1081_MOESM2_ESM.docx]

**Additional Table S2.** Relationship of TyG index (per-1 unit increase) with the progression of coronary plaque sub-types

|  | Fibrous | | Fibrous-fatty | | Necrotic-core | | Dense calcium | |
| --- | --- | --- | --- | --- | --- | --- | --- | --- |
|  | OR (95% CI) | P | OR (95% CI) | P | OR (95% CI) | P | OR (95% CI) | P |
| Model 1 | 1.462 (1.184−1.805) | <0.001 | 1.334 (1.091−1.630) | 0.005 | 1.354 (1.095−1.674) | 0.005 | 1.557 (1.213−1.998) | 0.001 |
| Model 2 | 1.250 (0.996−1.570) | 0.055 | 1.208 (0.971−1.503) | 0.089 | 1.179 (0.934−1.487) | 0.165 | 1.343 (1.027−1.758) | 0.031 |

BMI, body mass index; CI, confidence interval; OR, odds ratio; TyG index, triglyceride-glucose.

Model 1: Unadjusted

Model 2: Adjusted for age, male sex, hypertension, diabetes, hyperlipidemia, and BMI ≥25.0 kg/m^2^
