# Supplementary material for: Quantitative assessment of coronary plaque volume change related to triglyceride glucose index: The Progression of AtheRosclerotic PlAque DetermIned by Computed TomoGraphic Angiography IMaging (PARADIGM) registry
Source: Cardiovasc Diabetol. 2020 Jul 18;19:113. doi: 10.1186/s12933-020-01081-w (PMC7368987; doi:10.1186/s12933-020-01081-w)
Supplement: Supplementary file 3 — Additional file 3: Table S3. Association of clinical variables with the annual change of total PV, TAVnorm, and PAVtotal. [file 12933_2020_1081_MOESM3_ESM.docx]

**Additional Table S3.** Association of clinical variables with the annual change of total PV, TAV_norm_, and PAV_total_

|  | Annual change of  total PV | | Annual change of  TAV_norm_ | | Annual change of  PAV_total_ | |
| --- | --- | --- | --- | --- | --- | --- |
|  | β | p | β | p | β | p |
| Age, per 1 year | 0.549 | <0.001 | 0.614 | 0.002 | 0.020 | <0.001 |
| Male | 2.518 | 0.098 | 5.150 | 0.006 | 0.167 | 0.009 |
| Systolic BP, per 1 mmHg | 0.140 | 0.004 | 0.044 | 0.473 | 0.001 | 0.808 |
| Diastolic BP, per 1 mmHg | 0.156 | 0.040 | 0.011 | 0.912 | 0.002 | 0.252 |
| BMI, per 1 kg/m^2^ | 0.741 | 0.003 | 0.613 | 0.049 | 0.014 | 0.200 |
| Total cholesterol, per 1 mg/dL | 0.006 | 0.762 | -0.065 | 0.007 | -0.002 | 0.021 |
| HDL-C, per 1 mg/dL | -0.174 | 0.005 | -0.303 | <0.001 | -0.012 | <0.001 |
| LDL-C, per 1 mg/dL | -0.011 | 0.614 | -0.060 | 0.029 | -0.002 | 0.081 |
| TyG index, per 1 unit | 2.794 | 0.030 | 3.817 | 0.015 | 0.172 | 0.002 |

BMI, body mass index; BP, blood pressure; CI, confidence interval; HDL-C, high-density lipoprotein cholesterol; LDL-C, low-density lipoprotein cholesterol; PAV_total_, total percent atheroma volume; TAV_norm_, normalized total atheroma volume; TyG, triglyceride glucose.
